# Supplementary material for: Causal modelling demonstrates metabolic power is largely affected by gait kinematics and motor control in children with cerebral palsy
Source: PLoS One. 2023 May 24;18(5):e0285667. doi: 10.1371/journal.pone.0285667 (PMC10208503; doi:10.1371/journal.pone.0285667)
Supplement: S1 Appendix — (DOCX) [file pone.0285667.s002.docx]

S1 Appendix

**Table A: Adjustment sets for factors not discussed in the main text**

|  | Adjustment Set |
| --- | --- |
| Speed | GDI, DMC, SMC, spasticity, strength, age, height, mass |
| Age | N/A |
| Mass | Age, height |
| Height | Age |
| Sex | N/A |

*BART models for each factor include the factor and its adjustment set as input variables.

**Figure A.** Accumulated local effects plots depicting the total (mediated) effects of the factors not directly discussed in the main text. These plots represent the average change in metabolic power that can be expected with a change in the x-axis variable. X-axis variables are normalized as z-scores (excluding sex), where more positive scores indicate increasing mass, age, speed, and height. The bottom right scale indicates how large a single z-score is with respect to the original units of the factor. Rug plots along the x-axis display the distribution of scores for each factor (excluding sex). Included are 95% bootstrapped confidence intervals for all variables except sex.

**Figure B.** The range of the causal effect for each factor within the middle 95th percentile of the data. The dotted and dashed lines show the 25th percentile and median metabolic power of children included in this study. The total effects of age and mass are the largest. Height and speed have slightly smaller effects on metabolic power than age or mass. Note that the effects of age and height are mediated by mass, so their total causal effects also reflect mass-related changes in metabolic power due to aging and growth. The effect of speed indicates how metabolic power may change due to walking slower or faster. Sex has close to no effect on metabolic power in children with CP.
